# Active Commuting: Workplace Health Promotion for Improved Employee Well-Being and Organizational Behavior

**DOI:** 10.3389/fpsyg.2016.01994

**Published:** 2017-01-10

**Authors:** Nadine C. Page, Viktor O. Nilsson

**Affiliations:** Hult International Business SchoolBerkhamsted, UK

**Keywords:** workplace health promotion, active commuting, electric bikes, employee well-being, organizational behavior, business success

## Abstract

**Objective:** This paper describes a behavior change intervention that encourages active commuting using electrically assisted bikes (e-bikes) for health promotion in the workplace. This paper presents the preliminary findings of the intervention’s impact on improving employee well-being and organizational behavior, as an indicator of potential business success.

**Method:** Employees of a UK-based organization participated in a workplace travel behavior change intervention and used e-bikes as an active commuting mode; this was a change to their usual passive commuting behavior. The purpose of the intervention was to develop employee well-being and organizational behavior for improved business success. We explored the personal benefits and organizational co-benefits of active commuting and compared these to a travel-as-usual group of employees who did not change their behavior and continued taking non-active commutes.

**Results:** Employees who changed their behavior to active commuting reported more positive affect, better physical health and more productive organizational behavior outcomes compared with passive commuters. In addition, there was an interactive effect of commuting mode and commuting distance: a more frequent active commute was positively associated with more productive organizational behavior and stronger overall positive employee well-being whereas a longer passive commute was associated with poorer well-being, although there was no impact on organizational behavior.

**Conclusion:** This research provides emerging evidence of the value of an innovative workplace health promotion initiative focused on active commuting in protecting and improving employee well-being and organizational behavior for stronger business performance. It considers the significant opportunities for organizations pursuing improved workforce well-being, both in terms of employee health, and for improved organizational behavior and business success.

## Introduction

### Workplace Health Promotion

A healthy and productive workforce is critical for economic success and population health (Cancelliere et al., 2011). Unhealthy working adults contribute to a substantial economic burden of health-related productivity loss (Burton et al., 2004; Goetzel and Pronk, 2010; Eng et al., 2016). With regards to health, physical (in)activity directly contributes to one in six deaths and costs £7.4 billion to business and wider society (Petrokofsky and Davis, 2016). According to the World Health Organization (WHO), non-communicable diseases are estimated to have reduced the Gross Domestic Product by one percent in most low- and middle-income countries by 2015 (Abegun and Stanciole, 2006). Due to the expansion of sedentary occupations and an aging population, over a quarter of adults in England report to have less than 30 min of physical activity a week (Public Health England, 2014); this is significantly lower than the recommended amount (Department of Health, 2011). In light of this, the WHO has identified the workplace as one of the priority settings for health promotion in the 21st Century.

The workplace presents an ideal setting to promote and deliver health-promotion activities; overcoming barriers such as a lack of time and providing access to a large intersection of society (Malik et al., 2014). Workplace health promotion is important in the prevention of non-communicable diseases among employees. For example, workplace health promotion programs have shown to offer benefits by improving employees’ blood pressure levels (Eng et al., 2016), lowering disease prevalence (Boshtam et al., 2010; Jung et al., 2012), reducing stress (Jarman et al., 2015), lowering sickness absence (Loeppke et al., 2008; Goetzel et al., 2009), and improving presenteeism (Cancelliere et al., 2011). They have also been shown to produce happier, healthier, more loyal and productive employees (Fitzgerald and Danner, 2012). However, despite long-standing advocacy for comprehensive worksite programs, there needs to be more empirical evidence that links these strategies to improvements in health and productivity (Terry et al., 2008), and further consideration to the types of intervention that encourage behavior change and maintenance (Hunter et al., 2016). In essence, further implementation research is needed.

Recent National Institute for Health and Clinical Excellence (NICE) guidelines recommend the promotion of physical activity in and around the workplace, particularly through walking and cycling (National Institute for Health and Clinical Excellence [NICE], 2008). One way employees might be encouraged to be more active at work is by integrating active commuting modes such as walking and cycling into daily routines. This has been recommended as the best way of encouraging physical activity (Petrokofsky and Davis, 2016), and will be explored further in this study.

### Commuting Behavior

There is increasing interest in commuting behaviors for health and environmental benefits, especially against the backdrop of climate change and the global physical activity ‘pandemic’ (Kohl et al., 2012; Shaw et al., 2014). However, it seems that passive commuting modes remain the preferred option. Car use accounts for 76% of commuting behavior for journeys greater than two miles and active commuting modes such as walking and cycling account for just 3% (Department for Transport, 2007). As such, commuting behavior is one target area for health promotion; it presents an opportunity to shift, where appropriate, passive travel behavior (e.g., car use) to more active modes (e.g., walking, cycling) for improved environmental and personal well-being. A workplace health program focused on active commuting might help to change employee behavior and bring about broader benefits for the organization.

### Active Travel and Health

Active travel modes include walking or cycling as an alternative to motorized transport for the purpose of making everyday journeys. Encouraging people to switch their journeys to active travel modes can improve health, quality of life, and the environment (Woodcock et al., 2009), and will directly benefit individuals and communities as a whole. Integrating active commuting modes such as walking and cycling into daily routines is recommended as the best way of encouraging physical activity (Petrokofsky and Davis, 2016). Indeed, people who cycle for travel purposes, as opposed to leisure, are four times more likely to meet physical activity guidelines than those who don’t (Stewart et al., 2015). Cycling to work is very good exercise that is relatively easy to incorporate in normal daily routines (Vuori et al., 1994). Furthermore, the workplace setting is a logical place to promote active travel since in many countries the majority of adults work (OECD, 2014) and as stated above, a large proportion of journeys to work are made by private motor vehicles (Enoch and Rye, 2006; Goodman, 2013). Following on, this study describes a workplace behavior change intervention for encouraging active commuting.

### The Impact of Commuting

Commuting is an integral part of the workday routine; it is the connection between home- and work-life. Commute mode and commuting experience can have an impact on who we are, not just in terms of physical health, but with regards to our overall well-being and behavior (Santhosh, 2015). Further, these effects can manifest both at home and in the workplace, and in a bidirectional way: work and home life can seep into the commute in many ways and the commuting experience can seep back into work and home life (Bissell, 2015). Commuting to and from work has been found to create subtle but significant transformations in people over time, in relation to the way they interact, what they desire from work and home life, personal tolerance and coping levels, and habits in thinking and feeling (Bissell, 2015). In sum, an employee’s mode of commute and the commuting experience can have both a positive and negative effect.

### Commuting Experience

Experiences during the commuting journey can change what people are capable and willing to do at work. Indeed, as Leiter and Durup (1996) suggest, workplace attitudes and behaviors can be influenced significantly by non-work factors. Furthermore, unresolved daily hassles, such as those that might be experienced during the commute to work, can persist even when they are no longer in the conscious awareness and they can add to hassles in other life contexts (Kohn and Macdonald, 1992). Put differently, an employee’s commuting experience can spillover and influence their behavior and performance at work, and this might lead to decreases in productivity (see Regus International, 2012).

Research exploring the impact of commuting has focused in two areas: studies that have explored the negative impacts that are largely associated with long and stressful commutes and studies that have explored the positive impacts of an active commuting mode. Dealing with the former first, experiencing stress as a result of commuting is fairly commonplace. For example, the majority of Australian commuters are stressed as a result of their daily trips to and from work (IBM, 2011) and in the UK, traffic congestion was the most commonly reported source of daily stress among employees (BBC News, 2000). Longer commuting times and greater commuting distances have been associated with negative psychological and behavioral outcomes (Koslowsky et al., 1996), and poorer well-being (Stutzer and Frey, 2008). Women seem especially susceptible to the impact of long commutes (Evans et al., 2002).

A stressful commute to work can potentially lead to poorer cognitive, behavioral, and emotional performance at work (Schaeffer et al., 1988), and also associate with a range of physical and emotional health problems (Crabtree, 2010). For example, Hennessy (2008) found a negative spillover of the traffic environment on workplace behavior. The results of this study suggest that dealing with traffic stress on the commute to work might deplete coping resources and thereby make employees less able to deal with workplace stress (see also Leiter and Durup, 1996).

The same resource-based hypothesis could also explain poorer cognitive and behavioral performance. In sum, it seems that negative experiences on the journey to work can spillover and have a negative impact on employees’ emotional well-being and, in turn, broader workplace performance, and viz. business success.

### Commuting Mode

With regards to commuting mode, an active commute can associate with positive outcomes, especially those related to physical health (Petrunoff et al., 2016a,b). Research on the associations between active travel and health has focused on physical health outcomes (Amlani and Munir, 2010) including major diseases and mortality (Jarrett et al., 2012; Laverty et al., 2013) and overall well-being (Martin et al., 2014). Specifically, cycling as a means of active travel is associated with reduced absence at work due to any form of sickness (Hendriksen et al., 2010). The more often people cycle to work and the longer the distance traveled, the lower the absenteeism. In other words, cycling to work not only contributes to employee health, it may also result in a financial benefit for the employer (Hendriksen et al., 2010).

As Hendriksen et al.’s (2010) study suggests, empirical research in this area is moving toward a broader scope and recent findings have suggested that there might be broader benefits of an active commute for both individuals and organizations, including greater overall well-being (Gómez et al., 2013) and social inclusion (Woodcock et al., 2009). Specifically, Mytton et al. (2016) found positive associations of active commuting with physical and mental well-being and lower sickness absence for employees who maintained cycling to work. These associations warrant further exploration. The aim of this paper, therefore, is to describe the implementation of a workplace health program that encourages active commuting, and further explore the associations of active and passive commuting modes with employee well-being and the broader co-benefits on organizational behavior.

Specifically, one organizational behavior that might be negatively impacted by commuting experience and commuting mode is Organizational Citizenship Behavior (Organ, 1988). Organizational Citizenship Behavior, defined as “individual behavior that is discretionary, not directly or explicitly recognized by the formal reward system, and that is aggregate, promotes the effective functioning of the organization. …the behavior is rather a matter of personal choice, such that its omission is not generally understood as punishable” (Organ and Ryan, 1995). Organizational Citizenship Behavior has been described as one prominent variable that indicates the significant manpower in organizational effectiveness. Williams and Anderson (1991) identified two dimensions of OCB; behavior exhibited toward individuals (OCBI) and behavior toward organizations (OCBO). Luthans (2005) describes the dispositional traits of employees with OCB to be cooperative, helpful, caring and conscientious. Despite the theoretical association between workplace commuting experience and OCB, the empirical research has not consistently identified the existence or nature of the relationship (Santhosh, 2015). In light of these findings, this study explores the relationship between commuting mode – an active compared with a passive commute – and Organizational Citizenship Behavior as an organizational co-benefit.

### Electrical Assisted Bikes for Active Commuting

Active commuting using a conventional bicycle might not be a viable alternative to passive commuting for some employees. This might be because of the increasing distances between home and work – a maximum distance of 10 km to the workplace was found to be a feasible commuter cycling distance using a conventional bike (Iacono et al., 2010) – or because of the topography of certain urban environments and roads that are not adapted for cyclists. As well as these physical constrains, people also commonly cite a lack of time; a lack of bicycle facilities at the workplace; and poor physical fitness and age as factors that deter them from choosing the conventional bicycle for transport (de Geus and Hendriksen, 2015).

Electrical assisted bikes (e-bikes), also called pedelecs, are becoming an increasingly popular mode of transport for leisure and active commuting (Papoutsi et al., 2014; Peterman et al., 2016) and their use might help to challenge some of the barriers associated with cycling. E-bikes provide electric assistance only when a rider is pedaling. They have a sensor to detect the pedaling speed, pedaling force, or both, and are typically limited to a maximum speed of 25 km/h. They are more environmentally sustainable than using a car – they produce no emissions, no noise and they use very little energy at very low cost (Intelligent Energy Europe, 2012). They also make active travel a more viable alternative to using a conventional bicycle and reduce the problems associated with cycling on hilly terrains and longer distances (Sperlich et al., 2012). The commuting distance with an e-bike is 1.5 times longer than with a conventional bicycle (Hendriksen et al., 2008). The additional ‘assistance’ provided by the e-bike also provides new motivation for novice cyclists and increases the likelihood that those users will continue cycling in the future (de Geus and Hendriksen, 2015).

The desire for increased speed and reduced physical exertion is reported to be the main motivation for the increasing popularity of e-bikes (MacArthur et al., 2014; Johnson and Rose, 2015). There is growing interest in the role that e-bikes can play in promoting health, and despite some concerns that electrically assisted cycling might not contribute sufficiently to minimum physical activity requirements (Simons et al., 2009; Sperlich et al., 2012), there is mounting empirical evidence to suggest that cycling with assistance can confer positive health benefits (Gojanovic et al., 2011; Louis et al., 2012). There are direct positive physical health outcomes. Peterman et al. (2016) found that using an e-bike helped participants meet their physical activity recommendations and improve their cardiometabolic risk factors within only 4 weeks. Following their assessment of the physiological demands of pedaling an e-bike, Louis et al. (2012) concluded that electrically assisted cycling has great potential to promote physical activity in industrialized societies.

Evidence on the broader psychological and behavioral impacts of e-cycling is less clear, although some studies have reported an increased sense of enjoyment of the user (Popovich et al., 2014; Fyhri and Fearnley, 2015). Furthermore, Theurel et al. (2012) found that postmen performed better on a mail-sorting test after delivering mail on an e-bike compared with a bicycle without assistance. These positive results were probably due to the fact that participants using the e-bike were less exhausted and could concentrate better. Research on the use of e-bikes is growing and there is certainly scope for further research that explores the feasibility and benefits of using e-bikes for active commuting as part of a workplace health program.

### Changing Travel Behavior

There are clear health benefits of active travel for individuals and co-benefits for organizations. However, the cycling culture in the UK is small (1% of all trips per year; Jones et al., 2016) and there has been limited effectiveness of physical activity behavior change interventions (Abraham and Graham-Rowe, 2009; Conn et al., 2009; Rongen et al., 2013). Behavior change approaches can be broadly categorized as structural or psychological (Fujii and Kitamura, 2003). More specifically, their design can be grouped into four broad categories: (1) economic policies focused on changing the finance system; (2) physical policies focused on changing the infrastructure; (3) soft policies, which are focused on behavior change; and (4) knowledge policies that support research and development (Shaw et al., 2014). There is ongoing development in the UK to improve the infrastructure that supports active travel (e.g., Cycling Delivery Plan and the Cycling and Walking Investment Strategy) and this is supported by significant investment in electrically assisted cycle sharing schemes. However, for these schemes to be effective, people need to change their behavior toward active travel modes and this requires effective behavior change interventions.

Studies on travel behavior have suggested that multi-component interventions are often more effective in creating behavior change (Ferdowsian et al., 2010), and for inducing long-term change (Goetzel and Pronk, 2010). Furthermore, when encouraging walking and cycling behaviors as alternatives to using the car, Ogilvie et al. (2004) found that those interventions that were participatory, as opposed to informational, were found to be the most effective. Intensive interventions with individuals have also been shown as an effective method to increase walking and cycling (Yang et al., 2010).

Wider social and economic concerns with health and well-being have led to an increased number of workplace health interventions, and a related increase in research on their effectiveness. However, previous research has shown modest and short-term results of interventions to date (National Institute for Health and Clinical Excellence [NICE], 2008; Conn et al., 2009; Foster et al., 2012). There is a recognized need for more innovative approaches and interventions that purposefully encourage physical activity behavior change maintenance (Hunter et al., 2016) and more high-quality research to inform policy and practice in the UK and elsewhere (Carmichael et al., 2016). Therefore, this paper focuses on describing a workplace health behavior change intervention for encouraging active commuting using e-bikes, and reports on the preliminary findings of the intervention’s impact on employee and organizational well-being.

This study addresses the following research questions: *Are there differences in personal well-being and organizational behavior according to active and passive travel modes? What is the impact of active and passive commuting mode on personal well-being (physical and psychological health) and organizational behavior? What is the relationship between frequency and distance of commute and personal well-being and organizational behavior, and does this differ according to commuting mode? What are the perceived barriers to active commuting and do perceptions of these change following a change in commuting behavior?*

## Materials and Methods

### Research Design

The study is designed as a quasi-experimental longitudinal intervention designed to change workplace travel behavior, and adopts a mixed method approach. Both quantitative and qualitative data were collected from baseline (pre-intervention), throughout the intervention phase, and are planned post-intervention. This paper focuses on the quantitative data that were collected online via monthly questionnaires and weekly diaries. All data were self-reported. At the time of writing, the data collection was mid-way through the intervention phase, approximately 8 weeks into the intervention.

### Organization Location

This research was conducted at the UK campus of a global education provider. The campus is rurally located approximately 5 km from the nearest town and public transport links. This makes car-use the default travel mode for the majority of employees and because of the hilly terrain, e-cycling is a more viable alternative compared with conventional cycling.

### Participants

All employees of the case organization were eligible to participate. Participants formed a self-selecting sample and were naturally assigned, through self-selection, to either the active travel group (Active Travel) or the non-active (Passive Travel) group. At the start of the research, 12 participants were included in the Active Travel group and 19 participants affiliated with the Passive Travel group. Due to the longitudinal nature of the research, there was a degree of participation withdrawal/non-compliance over time. The size of the participant groups therefore varied at different time points (see **Table 1**). The majority of participants were female (80%) and were aged between 21 and 55 years. The age distribution was similar for the active and passive travel groups (21–25 years: *n* = 3, 5; 26–30 years: *n* = 2, 2; 31–35 years: *n* = 1, 3; 36–40 years: *n* = 1, 1; 41–45 years: *n* = 2, 5; 46–50 years: *n* = 0, 0; 51–55 years: *n* = 2, 2; 56–60 years: *n* = 1, 1, for active and passive travel groups, respectively). All participants were day-shift workers although their start time was specific to their role.

**Table 1:** Data collection throughout the workplace intervention.

	Active travel	Passive travel
Week 1	12	19
Week 2	11	11
Week 3	8	10
Week 4	7	8
Week 5	6	8
Week 6	6	6
Week 7	4	5
Week 8	2	2
Monthly pre survey	13	8
Monthly 1 month	9	7

*The active travel group also includes employees who cycled to work not using an e-bike. These participants were not included in the inferential analyses.*

### The Workplace Travel Intervention

The workplace behavior change intervention was multi-component. The initiative started with an e-bike taster session that was delivered during a lunchtime at the case organization approximately 1 month prior to the start of the intervention. The taster session offered all employees from across the organization the opportunity to trial a power-assisted electric bike (e-bike) for a 10-min guided cycle ride. Participants were introduced to the e-bikes, their functionality including the maximum mileage range and speed that was possible with a fully charged battery, and to how remove and charge the battery. During this session, the researchers were available to answer any questions about the e-bikes and provide further information about the intervention. As follow-up to this, all employees across the organization received an information email detailing the workplace travel intervention and were invited to participate.

Participants in the intervention (Active Travel) group were given the opportunity to borrow an e-bike, free of charge, for up to 5-months duration (from May to September). The timing of the research was selected intentionally to encourage behavior change; it was hoped that the milder weather would be less of a deterrent. There were 10 Giant Prime E+3 W e-bikes available for loan and these were allocated to employees on a first come, first serve basis following the e-bike lunchtime taster session and follow-up email invitation. The intervention started with an e-bike induction, which informed participants about the functionality of the e-bike and ensured they had sufficient cycling proficiency.

Participants in the Active Travel group were loaned the e-bike for as long as they requested. Rather than imposing a minimum or maximum loan period, we decided to leave this open-ended to enable participants to have complete control over how long they borrowed the e-bike. We hoped that having this flexible approach would encourage a broad range of employees to borrow an e-bike and try active commuting even if this was for just a few days or a week. The median loan period at the time for this paper was 6 weeks (minimum = 3 weeks; maximum = 8 weeks). This suggested that participants did in fact continue with their behavior change for a sustained period of time. In addition, we did not insist that participants used the e-bike every day for their commute; again, employees were in complete control of how often they used the e-bike to commute; this was determined by their personal schedule. Lastly, we did not restrict participants to only using the e-bike for their commute; they were able to use the e-bike for any journey they wanted to. In essence, we gave participants complete control over where, when, and for how long they used the e-bikes.

The e-bikes were supplied with a battery, battery charger and lock, and were delivered to participants’ home addresses or to their workplace, as requested. A similar process was followed when participants wanted to return the e-bikes; we arranged a convenient location and time to collect the e-bike.

During the e-bike loan period participants were provided with fully comprehensive roadside assistance. This enabled them to be ‘rescued’ if their e-bike broke-down. Fortunately, this service was not used. Furthermore, we also serviced each e-bike mid-way through each loan period to ensure its running efficiency and safety.

The e-bikes were re-distributed to participants throughout the duration of the intervention on a rotational basis. Once an e-bike was returned, it was serviced and then re-issued to another employee, again for an open-ended duration. This approach ensured that the maximum number of employees were able to use an e-bike and participate in the intervention.

Participants in the control (Passive Travel) group continued with their usual car travel behavior for the duration of the study.

### Measures and Data Collection

Data were collected at the individual (employee) level pre-intervention, during the intervention phase, and will be collected post-intervention, via online monthly questionnaires and weekly diaries. A link to the questionnaires was sent individually to participants via email on a weekly/monthly basis. If participants had not completed these surveys within 2 days, a follow-up reminder email was sent. Participants in both the intervention and control groups completed near identical questionnaires, at the same time points, using the same data collection method.

The monthly questionnaire consisted of three major parts: (1) demographic variables including gender and age, (2) information on organizational behavior and (3) information on personal well-being. We measured positive and negative organizational behavior using the Organizational Citizenship Behavior (OCB) and Counterproductive Workplace Behavior (CWB) scales, respectively, developed by Dalal et al. (2009). The items from both scales were randomized and presented as one scale containing 14 items. Participants were asked to consider how frequently (*1 = not at all*; *5 = a great deal*) they had performed the behavior stated in each item (e.g., *“Went out of my way to be a good employee”*; *“Talked badly about people behind their back”*) in the last 4 weeks.

We measured personal well-being using the Flourishing scale (Diener et al., 2009) and the short General Health Questionnaire (GHQ12; Goldberg et al., 1997). The Flourishing scale contained eight items. Participants were asked to consider how strongly (*1 = strongly disagree; 5 = strongly agree*) they had felt a particular way over the past month (e.g., *“I have been a good person and have lived a good life”*). The GHQ was used to measure participants’ general physical health in the previous month (e.g., *“Been able to concentrate on what you are doing?”*). Participants rated each item according to whether they had felt that way more or less than normal (*1 = not at all; 4 = more so than usual*).

The weekly diary also consisted of three major components: (1) information on workplace commute (e.g., frequency, duration of journey), (2) barriers to active commuting (e.g., what factors stopped e-bike users/deterred car users cycling in future weeks), and (3) impact of the commute on personal affect (e.g., feeling energized, tired) and organizational behavior (e.g., ability to connect with other people at work, willingness to help others). The analysis presented here focuses mainly on the quantitative data collected from the weekly diaries.

The research was conducted in accordance with the recommendations of the Organization’s Research Ethics Protocol, and the Organization’s Research Ethics Committee. All participants gave written informed consent in accordance with the Declaration of Helsinki to confirm their agreement to participate in the research. Furthermore, all participants were comprehensively insured to cover personal injury for the duration of the study.

## Results

### Commuter Journeys

The median frequency of the bike usage was 1–2 days per week. The median loan period at the time of writing this paper was 6-weeks, ranging from 3 to 8 weeks. The median length (in time) of the commute was similar between both travel groups indicating that both active and passive travel groups spent an equal amount of time commuting each day. The average commuting distance (in kms) was longer for the Passive Travel group compared with the Active Travel group, indicating that the control group had a longer distance to commute to work (see **Table 2** for a summary).

**Table 2:** Workplace commute descriptive statistics for travel groups.

	Active travel	Passive travel
Usage (Median days/week)	1–2	5
Loan period (Median weeks)	6 (Minimum 3–Maximum 8)	N/A
Distance (Mean in km)	10.31 (Minimum 5.63– Maximum 20.92)	17.08 (Minimum 5.63– Maximum 29.12)
Travel time (Median in min)	21–30	21–30

### Active vs. Passive Commuting

A MANOVA was conducted to analyze the differences between the Active and Passive commuting groups on the outcome measures collected in the weekly diaries – changes in Organizational Behavior, Positive Feelings and Negative Feelings – over time. Using Pillai’s Trace, the was an overall moderate main effect of commuting group on the reported behaviors and the feelings, *V* = 0.30, *F*(3,100) = 14, *p* < 0.01, $\eta_{p}^{2}$ = 0.30. However, there was no significant effect of time, *V* = 0.22, *F*(21,306) = 1.14, *p* = 0.30, $\eta_{p}^{2}$ = 0.08, and no significant interaction of group and time, *V* = 0.15, *F*(21,306) = 0.74, *p* = 0.74, $\eta_{p}^{2}$ = 0.05.

Separate Univariate ANOVA_S_ were used to follow up the main effect of travel group from the MANOVA; the ANOVA_S_ were conducted with Travel Group as the independent variable with two groups (Active vs. Passive) and Organizational Behavior, Positive Feelings and Negative Feelings as separate dependent variables. A Bonferroni correction was used due to the multiple-comparisons and the new suggested *p-*value was 0.017 (0.05/3). The ANOVA_S_ revealed significant main effects of commuting group (Active vs. Passive) on Organizational Behavior, *F*(1,102) = 26.08, *p* < 0.01, $\eta_{p}^{2}$ = 0.20 and Positive Feelings, *F*(1,102) = 32.33, *p* < 0.01, $\eta_{p}^{2}$ = 0.24. The Active Travel group indicated both more positive organizational behavior and positive feelings compared to the Passive Travel group (see **Table 3** for descriptive statistics). There was no statistically significant difference between the commuting groups (Active vs. Passive) on Negative Feelings at the Bonferroni-corrected *p*-value, *F*(1,102) = 4.89, *p* = 0.029, $\eta_{p}^{2}$ = 0.05, although the difference in means was in the expected direction.

**Table 3:** Descriptive statistics on the outcome measures according to travel group.

		Travel mode	
		Active	Passive
Weekly measures	Organizational behavior	17.10 (2.95)	14.82 (1.63)
	Positive affect	22.32 (4.74)	16.71 (3.57)
	Negative affect	9.17 (3.17)	10.43 (3.36)
Monthly measures	Organizational Citizenship Behavior	21.65 (4.53)	22.38 (3.76)
	Counter-productive Workplace Behavior	34.16 (3.87)	34.31 (2.03)
	Flourishing	44.38 (4.72)	43.80 (4.79)
	GHQ12	38.84 (4.16)	32.67 (6.08)

A further MANOVA was conducted to analyze the differences between the Active and Passive commuting groups on the outcome measures collected in the monthly survey – Organizational Citizenship Behavior (OCB), Counterproductive Workplace Behavior (CWB), Flourishing, and general physical health as measured by GHQ12. Using Pillai’s Trace, there was a significant main effect of Travel Group on physical health, as measured by GHQ12, *V* = 0.37, *F*(4,29) = 4.24, *p* < 0.01, $\eta_{p}^{2}$ = 0.37. A follow up ANOVA indicated that the Active Travel group reported statistically significantly higher GHQ12 scores compared with the Passive Travel group, *F*(1,32) = 12.65, *p* < 0.01, $\eta_{p}^{2}$ = 0.28. There were no additional statistically significant effects of group, time or interaction (see **Table 3** for descriptive statistics).

These results suggest that active commuting associates with more positive organizational outcomes and that employees who use an e-bike for their commute also perceive greater personal well-being with more positive feelings and better physical health according to GHQ12. At this stage of the intervention, the differences in organizational behavior and well-being do not seem to differ according to commuting mode.

### The Impact of Commuting Mode

As well as exploring the differences between active and passive commuting groups, we also explored the changes within travel groups (Active and Passive) over the course of the intervention to discover the impact of commuting mode on personal well-being and organizational behavior (see **Table 4**). Paired-samples *t*-test were conducted to compare the pre-intervention score to the most recently reported score on Organizational Behavior and Affect separately for the Active Travel and Passive Travel groups. The alpha level was Bonferroni corrected to account for multiple comparisons; the new *p*-value = 0.025 (0.05/2).

**Table 4:** Descriptive statistics on the outcome measures according to travel group pre- and mid-intervention.

		Pre-intervention	Mid-intervention
Active travel group	Organizational behavior	14.63 (1.68)	17.05 (1.57)
	Total affect	32.22 (4.67)	36.49 (5.76)
	Organizational Citizenship Behavior	21.75 (3.31)	21.56 (5.76)
	Counter-productive Workplace Behavior	34.42 (4.27)	33.89 (3.48)
	Flourishing	44.08 (3.92)	44.67 (5.52)
	GHQ12	38.00 (3.86)	39.67 (4.47)
Passive travel group	Organizational behavior	14.00 (1.60)	14.86 (0.84)
	Total affect	29.13 (7.06)	29.54 (4.52)
	Organizational Citizenship Behavior	22.75 (4.20)	22.00 (3.32)
	Counter-productive Workplace Behavior	33.63 (1.69)	35.00 (2.38)
	Flourishing	44.88 (4.16)	42.71 (5.41)
	GHQ12	29.63 (6.57)	35.71 (5.59)

For the Active Travel group, those employees who participated in the intervention and changed their behavior from passive to active commuting, there was a statistically significant increase, equating to a large effect, in reported Organizational Behavior, *t*(8) = -4.14, *p* < 0.01, *d* = 1.44. Participants in the Active Travel group reported more positive Organizational Behavior at this stage (mid-intervention) compared with pre-intervention. Furthermore, the Active Travel group also reported a significant increase in Affect from pre-intervention, *t*(8) = -2.4, *p* = 0.04, *d* = 0.92, although this did not remain statistically significant according the Bonferroni *p*-value. However, it is worth noting that the magnitude of the effect was large according to Cohen’s *d* conventions. There was not, however, a statistically significant difference between the pre- and mid/intervention scores on Organizational Behavior or Affect for the Passive Travel group, *t*(7) = -1.51, *p* = 0.17, *d* = 0.05; *t*(7) = -0.28, *p* = 0.79, *d* = 0.06, respectively.

Paired samples *t*-test were also conducted on the pre- and mid-intervention scores on the OCB, CWB, Flourishing, and GHQ12 scales. However, these did not identify any statistically significant differences over time for either of the travel groups. It is worth noting that these data were collected monthly and therefore we had fewer associated data points at this stage of the research.

Overall, the results from the paired sample *t*-tests of the weekly diaries indicated that there was a positive change in reported Organizational Behavior and Affect from pre-intervention to mid-intervention among the Active Travel group, and this was associated with a change in behavior from passive to active commuting.

Pearson correlations were conducted to analyze the relationship between e-cycling frequency and Organizational Behavior and Personal Well-being. The Pearson’s correlation indicated a moderate positive relationship between e-bike use and Organizational Behavior, *r* = 0.59, *p* < 0.01, and Positive Affect, *r* = 0.64, *p* < 0.01. Furthermore, a Pearson’s correlation analysis indicated a moderate significant negative correlation between e-bike use and Negative Affect, *r* = -0.55, *p* < 0.01. In sum, the more frequently participants cycled to work, the better they felt. Put differently, greater behavior change was associated with more positive outcomes.

The length of time spent commuting did not tend to have any significant correlation with the behavioral or affective outcome measures among the Active Travel group. However, interestingly, the time spent on commuting was weakly correlated with negative feelings among the Passive Travel group, *r* = 0.28, *p* < 0.05.

Furthermore, for both travel groups, positive and negative affect was significantly associated with organizational behavior. Pearson’s correlations indicated a significant moderate positive correlation between Positive Affect and Organizational Behavior for the Active Travel group, *r* = 0.69, *p* < 0.01, and a low correlation for the Passive Travel group, *r* = 0.39, *p* < 0.01. Furthermore, there was a significant moderate negative correlation between Negative Affect and Organizational Behavior for the Active Travel group, *r* = -0.62, *p* < 0.01, and a low significant negative relationship for the Passive Travel group, *r* = -0.38, *p* < 0.01.

The results of the Pearson’s correlations highlight the relationships between commuting experience and well-being. A more frequent active commute is associated with more productive organizational behavior, positive affect, whilst a longer car commute is associated with more negative and affect.

### Perceived Barriers to Active Commuting

The participants in the Active Travel group reported on the factors that had previously deterred them from changing their behavior and cycling to work. Prior to using the e-bike, the most frequently reported concerns were road safety (68%), poor weather (89%), and poor road conditions (53%). We explored whether perceptions of these concerns changed as a result of changing behavior and cycling to work. Of these three reported concerns, the behavior change intervention was significantly associated with reducing concerns about road safety, *x^2^*(1) = 22.38, *p* < 0.01, with an odds ratio of 0.04, and road condition, *x^2^*(1) = 4.49, *p* < 0.05, odds ratio = 0.29. However, poor weather still remained the biggest concern after the intervention. Furthermore, the effect of using an e-bike decreased the frequency of reported concerns with hills from 28 to 0%, which was significantly associated according to Fisher’s Exact Test, *x^2^*(1)** =** 11.02, *p* < 0.01, Cramer’s *V* = 0.45.

## Discussion

The purpose of this study was to describe a workplace behavior change intervention that encourages active commuting using e-bikes, and to explore the associations of active vs. passive commuting on personal well-being (psychological and physical) and organizational behavior. Based on these preliminary data, we report three main findings, which are broadly in line with our expectations.

First, we found that there were both direct personal benefits and organizational co-benefits of an active commute compared with passive commuting. Employees who changed their behavior and undertook an active commute reported more positive affect and more productive organizational behavior compared with employees who continued with a passive commute, and this was attributable to their behavior change from a passive to active travel mode (as indicated by the pre- and mid-intervention comparisons). Indeed, these results, which concur with previous research that compares active travel using a conventional bike with car travel (Hendriksen et al., 2010; Martin et al., 2014; Mytton et al., 2016; Petrunoff et al., 2016a,b), suggest that there are multiple co-benefits of an active commute beyond those associated with improved physical health and carbon reduction. Further, these findings concur with previous researchers who have explored the impact of workplace health programs (e.g., Jarman et al., 2015) but additionally, they suggest that encouraging active commuting is a viable way to get employees to be more physically active with associated benefits for employees and their employing organization.

The use of electric bikes to encourage active commuting amongst employees was novel and offers a new approach to workplace health promotion that brings together the expertise of transport and health researchers and organizational psychologists who have, historically, often worked in siloes (Ogilvie et al., 2004). Future research should harness together the expertise from these disciplines.

Second, we found a positive relationship between e-cycling frequency and the outcome measures; more frequent use of the e-bike was associated with more positive affect, and more positive organizational behavior. In contrast, a longer passive commute was associated with more negative outcomes. These findings demonstrate the cumulative positive effects of active commuting and suggest that workplace health programs should encourage and support employees to develop habitual patterns of active commuting behavior so that this travel mode becomes the default and embedded within daily routines. As the results of this study show, there are progressive positive effects of e-cycling both for individuals and their organization.

These results also have implications for human resource management (HRM) practices and the recruitment of employees for a healthy workplace and business success. The employees in our sample lived relatively close to their place of work (between 5 and 30 km), which was located in rural and secluded location. Active commuting was a feasible alternative to passive commuting, although perhaps not perceived this way with a conventional bike (see next paragraph). However, even with a relatively short commute, there were still emergent differences between the active and passive travel groups in this study. These differences are likely to be magnified for longer (and more stressful) commutes. HR managers should consider the potential implications of these findings for not only controlling the organization’s environmental impact (Deming, 1986), but for also ensuring a healthy workforce for improved business success. An employee’s commuting distance to work should, perhaps, be considered as part of the recruitment process of the employee lifecycle. Organizations should consider how their recruitment practices can cultivate a ‘greener,’ healthier, and more productive workforce.

Although the distance of our employees’ commutes was manageable using a conventional bike, employees had strong perceptions about the barriers of cycling to work, and these had deterred them from undertaking an active commute using a conventional bike. Previous research (see Page and Page, 2014) suggests that people often have inaccurate perceptions about their behavior and the factors that influence it, and this can determine their efforts toward behavior change and the associated success of behavior change interventions, especially if these are designed around the perspective of bounded rationality choice models and the subject utility model of behavior (e.g., the majority of ‘soft’ psychological interventions; Page, 2015). The results of this study suggest that a participatory approach to behavior change challenges these inaccurate assumptions and reduces their inertial effects.

The results of this study also suggest that priori to cycling to work, the active travel group reported many concerns about an active commute and these were barriers to behavior change. However, following a change in commuting behavior, many of these concerns had reduced or diminished completely despite there being no physical change in many of these factors; active commuters simply changed their perceptions about cycling to work. These results suggest that behavior change interventions that are participatory, address behavior directly, and get people to try new behaviors rather than encouraging people to think about changing their behavior, might be more effective (see Page and Page, 2014; Page, 2015). Indeed, this approach has been effective in this workplace intervention. It appears that the performance of different behaviors has challenged thinking and weakened some of the cognitive barriers that had previously prevented behavior change.

Lastly, based on the findings of this study we have learnt something about changing employees’ behavior as part of workplace health programs. We suggest that workplace health interventions that encourage physical activity as part of an employee’s existing daily routine might result in greater behavior change maintenance, as desired (see Hunter et al., 2016), compared with approaches that seek to develop novel behaviors. Further, we found that our approach to behavior change, which was innovative in terms of offering employees the use of e-bikes free of charge and allowing employees complete control over their behavior change, worked really well. We found that not imposing any specific rules about usage encouraged engagement and allowed employees complete control over their approach to behavior change. Lastly, the use of a multi-component intervention involving both informational and participatory behavior change approaches was also successful and concurs with previous research (Ferdowsian et al., 2010; Goetzel and Pronk, 2010).

### Summary

This study, as far as the authors are aware, is the first to explore the effectiveness of a behavior change intervention that encourages active commuting using e-bikes for health promotion in the workplace. Previous studies exploring the differences between active and passive commuting have focused on conventional cycling (see Petrunoff et al., 2016a,b). Furthermore, those studies exploring the benefits of e-cycling have focused on the physical health benefits to the individual (e.g., Louis et al., 2012; de Geus and Hendriksen, 2015; Jones et al., 2016). No studies, as far as the authors are aware, have explored the broader benefits to the individual’s psychological health or the co-benefits for the organization. In light of the positive preliminary findings of this study, there is certainly much scope for future research to further explore the use of e-bikes to encourage behavior change as part of workplace health promotion programs.

### Strengths and Weaknesses

Limitations of the current results are noted. This study evaluated a workplace behavior change intervention to promote active travel behavior within a real world setting, which is both a strength and a limitation. The research design was quasi-experimental. It did include a control group but assignment to commuting groups was by self-selection. This automatically introduces a degree of bias to the research design. The offering of e-bikes to encourage active commuting is novel, was popular, and offers a new dimension and greater flexibility to active commuting.

While the data in this study are self-reported, this is entirely appropriate for well-being (which depends on self-report) and appears unlikely to have resulted in important biases for other measures. Previous research has shown good agreement between self-reported and objective estimates of commuting behavior (Panter et al., 2014) and health indices (Ferrie et al., 2005). The sample size was small and biased toward aﬄuent, educated and predominately white-collar, and the workplace location was also rather unique so the findings may not be readily generalizable to other populations and workplace settings. Furthermore, the low response rate of employees during the latter stages of the intervention has a negative impact on the representativeness of the sample.

## Conclusion

Whilst acknowledging that the results presented in this article are preliminary – the research is still live and a work in progress – and based on a small sample, the results of this innovative workplace behavior change intervention study are promising and potentially powerful. Overall, the findings concur with previous researchers (e.g., Hennessy, 2008; Petrunoff et al., 2016a,b) and assert to the direct benefits and co-benefits of an active commuting experience. Furthermore, they suggest that active commuting using an e-bike is a viable alternative to passive commuting, and is an innovative way of encouraging behavior change and developing the health and well-being of employees and their organizations for improved business success.

## Ethics Statement

This research was approved by Ashridge/Hult Research Ethics Committee. All participants gave informed consent to participate. All participants were fully insured for the duration of the trial. They received training and were issued with safety equipment.

## Author Contributions

NP: wrote/edited all sections of the paper. VN conducted the data analysis and drafted the Results section of the paper, which was edited and finalized by NP.

## Conflict of Interest Statement

The authors declare that the research was conducted in the absence of any commercial or financial relationships that could be construed as a potential conflict of interest.
